# A survey of scale insects in soil samples from Europe (Hemiptera, Coccomorpha)

**DOI:** 10.3897/zookeys.565.6877

**Published:** 2016-02-17

**Authors:** Mehmet Bora Kaydan, Zsuzsanna Konczné Benedicty, Balázs Kiss, Éva Szita

**Affiliations:** 1 Plant Protection Institute, Centre for Agricultural Research, Hungarian Academy of Sciences, Herman Ottó u. 15 H-1022 Budapest, Hungary; 2 Çukurova Üniversity, Imamoglu Vocational School, Adana, Turkey

**Keywords:** Hypogeal scale insects, faunal surveys, Berlese

## Abstract

In the last decades, several expeditions were organized in Europe by the researchers of the Hungarian Natural History Museum to collect snails, aquatic insects and soil animals (mites, springtails, nematodes, and earthworms). In this study, scale insect (Hemiptera: Coccomorpha) specimens extracted from Hungarian Natural History Museum soil samples (2970 samples in total), all of which were collected using soil and litter sampling devices, and extracted by Berlese funnel, were examined. From these samples, 43 scale insect species (Acanthococcidae 4, Coccidae 2, Micrococcidae 1, Ortheziidae 7, Pseudococcidae 21, Putoidae 1 and Rhizoecidae 7) were found in 16 European countries. In addition, a new species belonging to the family Pseudococcidae, *Brevennia larvalis* Kaydan, **sp. n.** and a new species of Ortheziidae, *Ortheziola editae* Szita & Konczné Benedicty, **sp. n.** are described and illustrated based on the adult female stage. Revised keys to the adult females of *Brevennia* and *Ortheziola* are presented.

## Introduction

Several expeditions were organized since the 1950’s mainly within mainland Europe by the Hungarian Natural History Museum to collect snails, aquatic insects and soil animals (mites, springtails, nematodes, and earthworms). More recently, these studies were focused on the Balkan Peninsula and the Carpathian Region (Csuzdi et al. 2011; Dányi 2010; Kontschán 2010; Mahunka and Mahunka-Papp 2010; etc.). For these studies, a total of 2970 soil samples were collected from different habitats such as forest litter, moss, agricultural areas etc. in 16 European countries. Although visual sampling is a widely used method and often very effective for collecting scale insect species (Hemiptera: Coccomorpha), other collecting methods such as Berlese funnel and D-Vac are also useful as both provide plenty of scale insect species living in leaf litter, soil and under moss (Kozár 2004; Kozár and Konczné Benedicty 2007).

Scale insects are small, sap-sucking true bugs, sister to Aphidoidea, Aleyrodoidea and Psylloidea (Gullan and Martin 2009). Scale insect taxonomy is generally based on the microscopic cuticular features of the adult female which are paedomorphic, maturing in a juvenile form (Kosztarab and Kozár 1988). García et al. (2015) indicated that more than 8000 species have been described up to now. Among these are many agricultural pests (Miller and Davidson 1990) and invasive species (Miller et al. 2005, Ouvrard et al. 2013).

It has been argued (Koteja 1985) that the evolution of the scale insects occurred in two stages. In the first stage, the scale insects split from the homopteran stock (in the Carboniferous or Permian) prior to the appearance of flowering plants (Jurassic), living in the forest litter on a “mixed” diet and feeding on the sap of various plants at the surface and from living and decaying plant tissues. The legs became modified as a digging organ (one claw, one segmented tarsus, functional tibiotarsus), the females lost their wings and became paedomorphic and the males became dipterous. They also diverged into numerous groups at that time. The second evolutionary trend commenced with the appearance of the flowering plants in the Jurassic and continues to the present. As a result of these latter trends, the coccoids became true plant parasites and most scale insect groups started to live on the aerial parts of the plants and acquired their own endosymbionts (Koteja 1996). As a result, the level of specialization in the recent scale insects is great although some species still continue the primary, hypogeal mode of life, e.g. in the families Ortheziidae (Nipponortheziinae, Newsteadinae, Ortheziolinae) and Rhizoecidae (Koteja 1986; Vea and Gimaldi 2012).

Our knowledge on the scale insect fauna of European countries is very variable (García et al. 2015; Kozár et al. 2013b). Despite the great heterogenity of habitat types and the zoogeographical importance of the area due to climate change, none of the countries of Europe could be considered as being well explored. Several investigations have been published on the economically important species (Argyriou et al. 1976; Masten Milek et al. 2008; Masten Milek and Simala 2008b; Masten Milek and Simala 2009; Santas 1989; Tomov et al. 2009; Trencheva et al. 2009; Trencheva et al. 2010), but much less attention has been paid to the native scale insect fauna living in natural habitats. The countries from which most species have been recorded are: France (381 species – Foldi 2001), Italy (390 species – Pellizzari and Russo 2004), Hungary (274 species – Kozár et al. 2013b), Bulgaria (145 species – Trencheva et al. 2012), Romania (207 species – Fetykó et al. 2010) Croatia (132 species – Masten Milek and Simala 2008a; Schmidt 1956; Zak-Ogaza 1967); and Greece (207 species – Pellizzari et al. 2015).

Although the pest scale insect species found on the aerial parts of agricultural and horticultural plants are well studied in Europe, there is a great gap in the knowledge on the hypogeal scale insect fauna in Europe. The aim of this study was to investigate the hypogeal scale insect fauna of Europe by studying the scale insect specimens found in the soil and litter samples of the Acarology Collection of HNHM, because hypogeal species are indicators of the ecological richness and biodiversity of the soils and provide useful information about the comparative ecologies of the regions, and about the evolution of soil animals.

## Material and methods

The specimens described and recorded in this study were all obtained from the soil samples in the Hungarian Natural History Museum (HNHM) collection (2970 samples in total). The samples were extracted by Berlese funnel. This is an apparatus widely used to extract living organisms, particularly arthropods. It works by creating a temperature gradient over the sample such that mobile organisms will move away from the higher temperatures and fall into a collecting vessel, where they are preserved for examination (Southwood and Henderson 2000). The Berlese funnel is a suitable device with which to collect and sort hypogeal and ground-dwelling animals, and also those which live in the lower herb layer of different habitats. It will also occasionally collect species living on higher aerial parts of plants that have fallen to the ground on plant material, such as leaves, twigs, etc.

Specimens were prepared for light microscopy using the slide-mounting method discussed by Kosztarab and Kozár (1988). The morphological terminology used follows Kozár (2004), Kozár et al. (2013a), Kozár and Konczné Benedicty (2007), and Williams (2004).

All measurements and counts were taken from all the available material, and the values are given as a range for each character.

Holotypes of the new species are deposited in the Hungarian Natural History Museum (HNHM). Paratypes are deposited in the HNHM and in the Plant Protection Institute, Centre for Agricultural Research, Hungarian Academy of Sciences (PPI).

Detailed locality and collection data have been provided for the new and some rare species only. For a host plant list of each species see García et al. (2015). Distribution data for each species have been provided, with new country records in bold. However must take into consideration, that these new country records are all relative to García et al. (2015) and latest available checklists (Fetykó et al. 2010; Masten Milek and Simala 2008a; Pellizzari et al. 2015; Trencheva et al. 2012), as to create new country checklists is out of the scope of this work.

## Results and discussion

Among 2970 soil samples, 280 samples (approximately 10%) contained scale insect specimens. Of these, 4 species are Acanthococcidae, 2 are Coccidae, 7 are Ortheziidae and 7 are Rhizoecidae, 21 are Pseudococcidae and there was 1 species of Micrococcidae and Putoidae. One new pseudococcid, namely *Brevennia larvalis* Kaydan, sp. n. and one new species of Ortheziidae, *Ortheziola editae* Szita & Konczné Benedicty, sp. n. are described and illustrated based on the adult female stage.

### Acanthococcidae

#### Anophococcus insignis

Taxon classificationAnimaliaHemipteraAcanthococcidae

Newstead

##### Material examined.

Croatia: 1 ♀ – Njivice.

##### Distribution.

United States of America, Armenia, Austria, Bulgaria, former Czechoslovakia, Denmark, France, Germany, Hungary, Iraq, Italy, Kazakhstan, Netherlands, Norway, Poland, Romania, Russia, Sicily, Sweden, Ukraine, United Kingdom (Channel Islands, England, Scotland) (García et al. 2015); **Croatia**.

#### Kaweckia glyceriae

Taxon classificationAnimaliaHemipteraAcanthococcidae

(Green)

##### Material examined.

former Czechoslovakia: 2 ♀♀ – unknown locality.

##### Distribution.

Austria, China, former Czechoslovakia, France, Germany, Hungary, Italy, Kazakhstan, Latvia, Poland, Romania, Russia, South Korea, Ukraine, United Kingdom (England), former Yugoslavia (García et al. 2015).

#### Pseudochermes fraxini

Taxon classificationAnimaliaHemipteraAcanthococcidae

(Kaltenbach)

##### Material examined.

Serbia: 2 ♀♀ – Braničevo District, Homoljske planina, Žagubica.

##### Distribution.

Austria, Belgium, Bulgaria, China, Croatia, Czech Republic, Denmark, Finland, France, Germany, Greece, Hungary, Iran, Italy, Lithuania, Luxembourg, Netherlands, Norway, Poland, Portugal, Romania, Russia, Spain, Sweden, Switzerland, Turkey, Ukraine, United Kingdom (England, Wales), former Yugoslavia (García et al. 2015); **Serbia**.

#### Rhizococcus reynei

Taxon classificationAnimaliaHemipteraAcanthococcidae

(Schmutterer)

##### Material examined.

Croatia: 2♀♀ – Njivice.

##### Distribution.

Germany, Hungary, Iran (García et al. 2015); **Croatia**.

### Coccidae

#### Lecanopsis turcica

Taxon classificationAnimaliaHemipteraCoccidae

(Bodenheimer)

##### Material examined.

Greece: 1 ♀ – Florina regional unit, Lehovo village.

##### Distribution.

Armenia, Cyprus, Georgia, Greece, Hungary, Romania, Russia, Slovenia, Turkey, Ukraine, former Yugoslavia (García et al. 2015).

#### Luzulaspis dactylis

Taxon classificationAnimaliaHemipteraCoccidae

Green

##### Material examined.

Romania: 1 ♀ – Harghita County, Praid (Parajd).

##### Distribution.

Czech Republic, Germany, Greece, Italy, Poland, Russia, Slovakia, United Kingdom (England) (García et al. 2015); Romania (Fetykó et al. 2010).

### Micrococcidae

#### Micrococcus confusus

Taxon classificationAnimaliaHemipteraMicrococcidae

Miller & Williams

##### Material examined.

Greece: 2 ♀♀ – West Greece, Aetolia-Acarnania regional unit, Akarnania Mts., Trifos village.

##### Distribution.

Algeria, Greece, Morocco (García et al. 2015).

### Ortheziidae

#### Arctorthezia cataphracta

Taxon classificationAnimaliaHemipteraOrtheziidae

(Olafsen)

##### Material examined.

Bulgaria: 1 ♀ – Borovets; 2 ♀♀ – Rila Mts., Struma basin, Rilomanastirska Gora Reserve, Stream Djavolska. Slovakia: 2 nymphs – Low Tatras, Stare Hory; 1 ♀ – 2 nymphs – Mutne; 3 nymphs – Pieniny Natural Park, Červený Kláštor; 1 nymph – Slovenský Raj NP, Vel’ký Sokol gorge, Kamenné vráta. Sweden: 2 ♀♀ – unknown locality.

##### Distribution.

Austria, Belgium, Canada, Corsica, Croatia, Czech Republic, Faeroe Islands, Finland, France, Georgia, Germany, Iceland, Ireland, Italy, Norway, Poland, Romania, Russia, Spain, Sweden, Switzerland, United Kingdom (England, Scotland), United States of America (García et al. 2015).

#### Arctorthezia helvetica

Taxon classificationAnimaliaHemipteraOrtheziidae

Kozár & Szita, 2015

##### Material examined.

Albania: 2 nymphs –Leskovik. Greece: 2 nymphs – Epirus, Ioannina regional unit, Melia village; 1 ♀ – Larissa regional unit, Ossa Mts.; 2 nymphs – West Greece, Aetolia-Acarnania regional unit, Kamaroula village. Serbia-Montenegro: 1 nymphs – Raška District, Pazariste village.

##### Distribution.

Switzerland (Szita et al. 2015); **Albania, Greece, Serbia**.

#### Newsteadia floccosa

Taxon classificationAnimaliaHemipteraOrtheziidae

(De Geer)

##### Material examined.

Albania: 1 ♀ – Has District, Pashtrik Mts., Salghinë village; 1 ♀ –Leskovik; 1 ♀ –Malësi District, Qafa e Valbones; 1 ♀ – Kukës District, Mali i Gjalica e Lumës; 1 ♀ – Shkodër District, Prokletije Mts., Kir village. Bosnia-Herzegovina: 1 ♀ – Ozren Mts., Vilić; 1 ♀ – Sutjeska valley. Bulgaria: 2 ♀ – Borovetz; 3 ♀ – Rodope Mts., Musala; 1 ♀ – Sinemorec; 1 ♀ – Stara Planina, Stidovska Mts. Croatia: 1 ♀ – Ivanšćica; 1 ♀ – Krk Island, Glavotok; 1 ♀ – Psunj Mts., Sisak-Moslavina county, Novska; 1 ♀ – Rab Island. Greece: 1 ♀ – Arcadia regional unit, Korfes village; 1 ♀ – Arkadia regional unit, Elliniko; 1 ♀ – Central Greece, Evrytania regional unit, Anatoliki Fragkista village; 1 ♀ – Epirus, Ioannina regional unit, Melia village; 1 ♀ – Florina regional unit, Verno Mts., Pisoderi village; 1 ♀ –Ioannina regional unit, Metsovo; 1 ♀ – Larisa regional unit, Ossa Mts.; 1 ♀ – Messinia regional unit, Haravgi, Polilimnio village; 1 ♀ – Thesprotia regional unit, Vrosina. Macedonia: 1 ♀ – Vinica Municipality, Obozna Planina Mts., Laki. Romania: 1 ♀ – Bihor County, Vlădeasa, Săcuien; 1 ♀ – Bihor County, Bihor Mts., Cetătile Rădesei; 1 ♀ – Bukovina County, Iedu; 1 ♀ – Bukovina County, Stratioara; 2 ♀♀ – Bukovina County, Valea Stânei; 1 ♀ – Caraş-Severin County, Semenic Mts., Văliug; 1 ♀ – Caraş-Severin County, Semenik Mts., Gărâna; 1 ♀, 1 nymph – Cluj County, Havasrekettye; 1 ♀ – Harghita County, Kis Beszterce; 1 ♀ – Harghita County, Sâncrăieni (Csíkszentkirály); 1 ♀ – Harghita County, Băile Homorod (Homoródfürdő); 1 ♀ – Harghita County, Băile Tuşnad (Tusnádfürdő); 3 ♀ – Maramureş County, Maramureşului Basin, Rona de Sus (Rónaszék); 1 ♀ – Maramureş County, Rodna Mts., Sǎcel (Izaszacsal); 1 ♀ – Maramureş County, Maramureş Mts., Vişeu de Sus; 1 ♀ – Maramureş County, Ignis, Mts., Plesca village; 1 ♀ – Maramureş County, Ignis Mts., Kőhát, Săpânța (Szaplonca); 1 ♀ – Maramureş County, Gutin Mts., Breb (Bréb); 1 ♀ – Maramureş County, Baia Mare (Nagybánya), Valhani plateau, Rozsály Mt.; 1 ♀ – Maramureş county, Maramureş Mts., Borşa-Băile Borşa, Vinişor valley; 1 ♀ – Oltenia, Leleşti; 2 ♀ – Oltenia, Runcu; 1 ♀ – Oltenia; Poiana Mărnlui; 1 ♀ – Sibiu District, Bradu (Fenyőfalva); 1 ♀ – Sibiu District, Cisnădioara (Kisdisznód); 1 ♀ – Hunedoara County, Petroşani (Petrozsény); 1 ♀ – Hunedoara County, Obersia. Serbia-Montenegro: 1 ♀ – Kosovo, Novo Selo; 1 ♀ – Maljen Mts., Ražana; 1 ♀ – Savino Polje, Đalovica klisura; 3 ♀♀ – Vojnik Mts., Mokro, Šavnik; 4 ♀♀ – Žabljak Municipality, Durmitor National Park, Crno Jezero. Slovakia: 1 ♀ – Becherov, Nizke Beskydy); 1 ♀ – Úhorná; 2 ♀ – Javorina (Jávoros); 1 ♀ – Košice District, Smolník; 1 ♀ – Liptovsky Osada; 4 ♀♀ – Slovenský Raj National Park; 1 ♀ – Stratehná; 1 ♀ – Tatranska Poliana; 1 ♀ – Závadka, Hronom Muvaska Plania. Slovenia: 1 ♀ – Triglav National Park, Koča pri Peričniku. Sweden: 1 ♀ –Hagfors; 4 ♀ – Ilsbo; 2 ♀ – Lapland Prov., Kiruna; 1 ♀ – Lysvik. Turkey: 1 ♀ – Kuru, Kuru Mts.

##### Distribution.

Austria, Belgium, Bulgaria, Corsica, Croatia, Czech Republic, Denmark, Finland, France, Germany, Hungary, Ireland, Italy, Lithuania, Netherlands, Poland, Romania, Russia, Spain, Sweden, United Kingdom (England, Scotland) (García et al. 2015).

##### Comments.

*Newsteadia floccosa* is the most common species in the collection. Although there is some variability in the number of antennal segments and in the size of the individuals examined in this study, all specimens above are considered to be part of the morphological variation of *Newsteadia floccosa*.

#### Newsteadia susannae

Taxon classificationAnimaliaHemipteraOrtheziidae

Kozár & Foldi

##### Material examined.

Albania: 1 ♀ – Sarandë District, Borsh; 1 ♀ – Tepelenë District, Griba Mts., Bënçë. Greece: 1 ♀ – Ioannina regional unit, Kalpaki, Vellas Monasteri. Serbia: 1 ♀ – Đerdap Mts., Mosna.

##### Distribution.

France (Corsica), Greece (Kozár 2004); **Albania, Serbia**.

##### Comments.

*Newsteadia susannae* is closest to *Newsteadia floccosa* but differs (i) in having hair-like setae on most antennal segments; (ii) a higher number of quadrilocular pores on venter and dorsum, and (iii) complete wax plate bands on mid dorsum (Kozár 2004).

#### Ortheziola

Taxon classificationAnimaliaHemipteraOrtheziidae

Šulc, 1895

##### Type species.

*Ortheziola vejdovskyi* Šulc, 1895, 1.

##### Diagnosis of genus.

**Figure 1.**
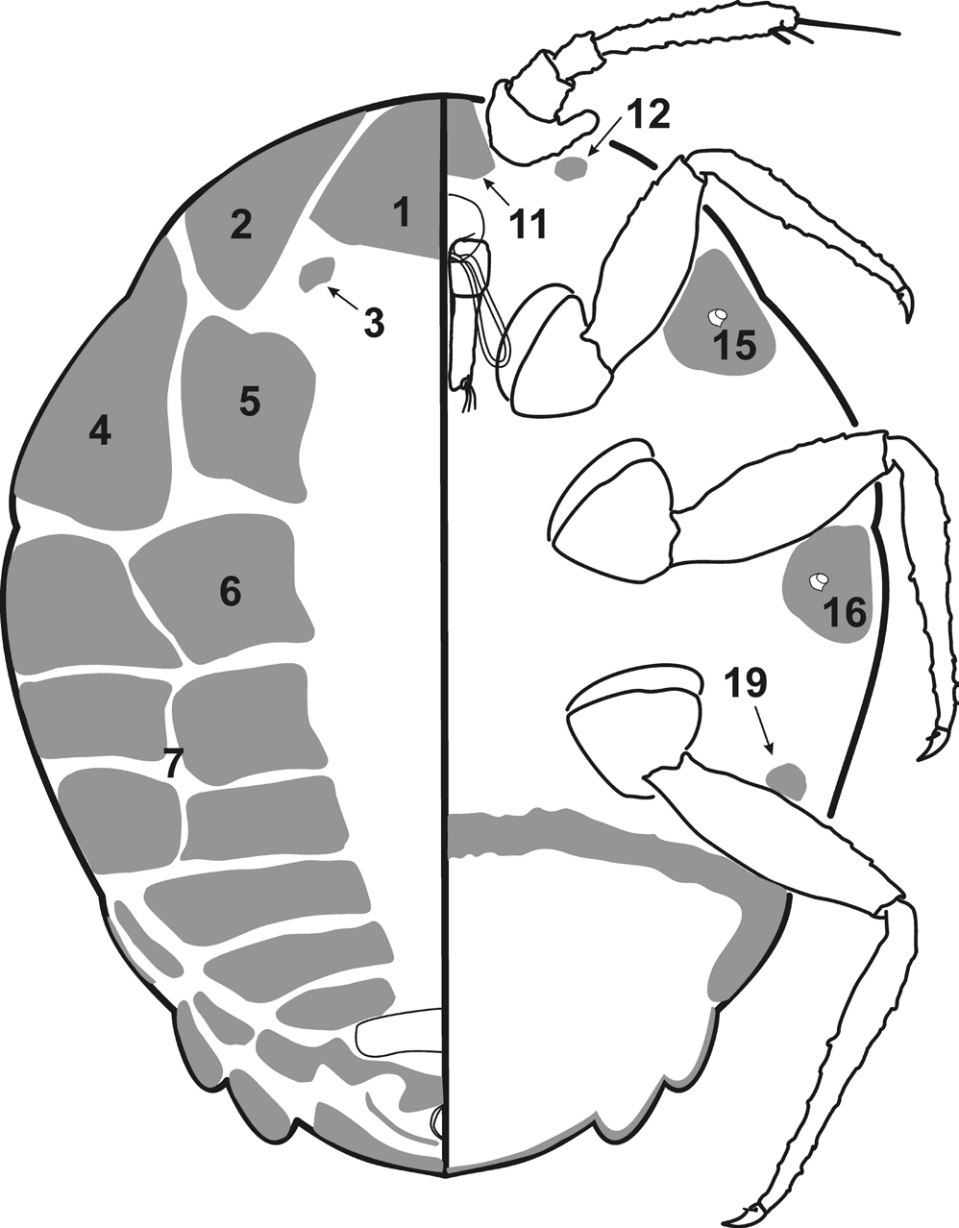
Distribution of waxplates in *Ortheziola* genus. Figure based on *Ortheziola britannica* Kozár & Miller, female; after Kozár 2004.

Adult female in life with a series of marginal, mediolateral and medial waxy protrusions, corresponding to wax plates on slide-mounted specimens. The distribution of these protrusions and wax plates (Fig. 1) differs between species in the genus (Kozár 2004).

Slide-mounted adult female with three-segmented antennae; third antennal segment with a slender apical seta, flagellate sensory seta and small subapical seta; second segment with one sensory pore. Eye stalk protruding, thumb-like, fused with sclerotized area at base of each antenna (sometimes called the pseudobasal antennal segment). Legs well developed; leg setae robust, spine-like; trochanter and femur fused, tibia and tarsus fused; tibia with one sensory pore and at least one fleshy sensory seta; tarsus without digitules; claw digitules hair-like, claw without a denticle. Labium one-segmented, with many setae; labium with three long setae near apex, very close together, all situated in a single setal socket . Anal ring situated in a dermal fold on dorsal surface, ring bearing six setae. Sclerotized plate present on dorsum anterior to anal ring, wider than long. Modified pores, each with two, three or four loculi, scattered over surface, appearing like microtubular ducts. Thumb-like pores forming a cluster on each side of anal ring. Abdominal spiracles ventral on anterior segments, with at least one present on each side of segments I, II or III; when present, posterior abdominal spiracles located on dorsum near anal ring, surrounded by a cluster of multilocular pores (Kozár 2004).

##### Distribution.

The 13 species of *Ortheziola* are found in the Palaearctic and north-eastern part of the Oriental Regions. For detailed distribution data of the twelve previously known species, see ScaleNet (García et al. 2015). New locality records for several *Ortheziola* species were discovered during the study of the HNHM collection, which is listed below. The distribution patterns of the species may imply the existence of several other species in these regions, which would be worth further study.

##### Comments.

The genus *Ortheziola* resembles the genera *Ortheziolacoccus* and *Ortheziolamameti* in having three-segmented antennae, with the basal part of the antenna fused to the eye. However, *Ortheziola* differs from *Ortheziolacoccus* and *Ortheziolamameti* in having only a single spine band inside the ovisac band, and these genera have different geographic distribution: *Ortheziola* species are distributed in the Palaearctic and north east part of Oriental Regions, *Ortheziolacoccus* species occur only in Ethiopian Region, while *Ortheziolamameti* species in the Oriental and Ethiopian Regions.

##### Key to species of *Ortheziola*, based on adult females

**Table:** 

1	Dorsal wax plates 5 and 6 present, either fused or separate	**2**
–	Dorsal wax plates 5 and 6 absent	**11**
2	Dorsal wax plate 3 present (represented by at least a small spine group)	**3**
–	Dorsal wax plate 3 absent	**12**
3	Dorsal wax plates 5 and 6 fused with marginal spine bands	***Ortheziola matskasii* Kozár & Konczné Benedicty**
–	Dorsal wax plates 5 and 6 clearly separate from marginal spine band	**4**
4	Dorsal wax plate 3 reduced to a small spine group	**5**
–	Dorsal wax plate 3 fully developed	**7**
5	Ventral plate 19 present; anterior margin of ovisac band almost completely straight	***Ortheziola britannica* Kozár & Miller**
–	Ventral plate 19 absent; anterior margin of ovisac band with characteristic waves	**6**
6	Anterior margin of ovisac band with at least 8 waves; several multilocular pores present anterior to vulva	***Ortheziola marottai* Kaydan & Szita**
–	Anterior margin of ovisac band with six waves; one or two multilocular pores present anterior to vulva	***Ortheziola editae* sp. n.**
7	Multilocular pores present around vulva	**8**
–	Multilocular pores absent from around vulva	**9**
8	Multilocular pores present both anterior and posterior to vulva; dorsal 5-locular pores present throughout the last three abdominal segments	***Ortheziola szelenyii* Konczné Benedicty & Kozár**
–	Multilocular pores present only anterior to vulva; dorsal 5-locular pores concentrated around anal ring	***Ortheziola vejdovskyi* Šulc**
9	Ventral wax plates 11 and 19 present	***Ortheziola peregovitsi* Kozár & Konczné Benedicty**
–	Ventral wax plates 11 and 19 absent	**10**
10	Ventral wax plate 12 present; marginal wax plates on abdominal segments IV-VI clearly separated from each other and from medial plates	***Ortheziola hauseri* Konczné Benedicty & Kaydan**
–	Ventral wax plate 12 absent; marginal wax plates on abdominal segments IV-VI fused to each other and partly fused to medial plates	***Ortheziola mizushimai* Tanaka & Amano**
11	Ventral wax plates 11 and 12 present, longest seta on antenna ca. 10 µm long	***Ortheziola viti* Konczné Benedicty & Szita**
–	Ventral wax plates 11 and 12 absent; shortest seta on antenna ca. 19 µm long	***Ortheziola marginalis* Kozár & Konczné Benedicty**
12	Multilocular pores present around vulva	***Ortheziola vietnamiensis* Kozár & Konczné Benedicty**
–	Multilocular pores absent from around vulva	***Ortheziola fusiana* Shiau & Kozár**

#### Ortheziola editae

Taxon classificationAnimaliaHemipteraOrtheziidae

Szita & Konczné Benedicty sp. n.

http://zoobank.org/7098E617-0BD8-4927-B36E-3DC3DC4C9E7A

Fig. 2

##### Material examined.

*Holotype*. Adult female. Bulgaria: Blagoevgrad province, Pirin Mts., Pirin, hazel bush towards Beljata Reka, N 41°35.968', E 23°32.809', 1280 m a.s.l., 26.x.2013, leg. Kontschán, Murányi, Szederjesi, litter and soil (PPI: 11912, HNHM: E-3079). *Paratypes*. Bulgaria: 3 ♀♀ on two slides: same data as holotype. *Other material examined*. Croatia: 1 ♀ – Papuk Mts., Drenovac, riverbank, 21.iv.2004, leg. Kontschán (PPI: 11911, HNHM: E-1864).

##### Diagnosis.

##### Description.

*Unmounted adult female*. Not seen.

*Slide mounted adult female*. Body 1.5–2.0 mm long, 1.2–1.3 mm wide. Length of antennal segments: 1^st^ 76–89 µm; 2^nd^ 46–56 µm; 3^rd^ 250–270 µm; 3^rd^ segment parallel sided or weakly clubbed; apical seta 127–173 µm, subapical seta 30–46 µm; fleshy sensory seta near apical seta 28–31 µm; basiconic sensilla present near apex of antenna; all segments of antennae covered with moderate number of robust spine-like, straight, apically acute setae, longest seta 15 µm long.

*Venter*. Labium 120–148 µm long. Stylet loop about as long as labium. Leg segment lengths: front coxa 107–127 µm, middle 117–133 µm, hind 122–127 µm; front trochanter-femur 291–332 µm, middle 321–357 µm, hind 316–362 µm; front tibia-tarsus 357–372 µm, middle 357–388 µm, hind 438–454 µm; front claw 46–54 µm, middle 43–51 µm, hind 51–54 µm long; claw digitules spine-like, 7–12 µm long; legs with rows of robust setae; longest seta on trochanter-femur, each 12–14 µm long; with one flagellate sensory seta on each of femur and tibia, 10–12 µm long; each trochanter with four sensory sensilla on each surface. Wax plate 11 and 12 present at marginal areas of head; marginal wax band surrounding each thoracic spiracle (plates 15 and 16); wax plates in front of coxae absent (plates 13, 14, 17 and 18 absent), plate 19 absent; with scattered clusters of spines between hind legs and ovisac band. Anterior margin of ovisac band with three waves; with one band of spines within ovisac band, with quadrilocular pores predominant near anterior edge of spine bands and scattered within the spinebands, each pore 3.5–4 µm in diameter. Thoracic spiracles each with scattered quinquelocular pores loosely associated with spiracle opening, each group contains 10–13 pores, each pore 5–6 µm in diameter (several of these pores present on dorsum); diameter of opening of anterior thoracic spiracle 13–20 µm. Setae few, scattered in medial areas of thorax, with several setae present near anterior margin of ovisac band (some capitate), several associated with anterior and posterior multilocular pore rows, several more associated with posterior multilocular pores surrounding vulva. Multilocular pores each 8–9 µm in diameter, with 7–9 (mainly 7) loculi around perimeter and one loculus in central hub; partial band of multilocular pores near anterolateral edge of spine band, also scattered around vulva and near ovisac band, almost forming a row on the apical abdominal segment. Abdominal spiracles present, two pairs on each side of body anterior to ovisac band and one pair situated inside ovisac band, near anterolateral angle; each abdominal spiracle with sclerotized vestibule.

*Dorsum*. Wax plates covering two-thirds of marginal area; mediolateral thoracic plates (3, 5 and 6) present; waxplate 3 small, containing only a few spines and pores; medial area of thorax and abdomen with a few scattered spines and pores. Spines at margin of wax plate 4 each 15–16 µm long, those in middle of wax plate each 16–18 µm long; spines truncate and expanded at apex. Flagellate setae present in very small numbers on each wax plates and in medial bare area, each seta 17 µm long. Quadrilocular pores, each 3.0–3.5 µm in diameter, with four loculi, present at the margins of all waxplates and scattered within the waxplates. Quinquelocular pores, each 5.5–6.0 µm in diameter, present in marginal areas of abdomen, between the waxplates; also present in a cluster near anal ring. Sclerotized plate on abdomen 63–77 µm long, 230–251 µm wide; with a few setae with pointed apices situated at posterior edge of plate. Anal ring with incomplete triple rows of circular pores, each pore 1.5–3.0 µm in diameter; longest anal ring seta 72–74 µm long; anal ring 60–67 µm long, 50–55 µm wide. Thumb-like pores, each 6 µm long. Abdominal spiracle present in center of multilocular pore cluster situated laterad to anal ring.

**Figure 2.**
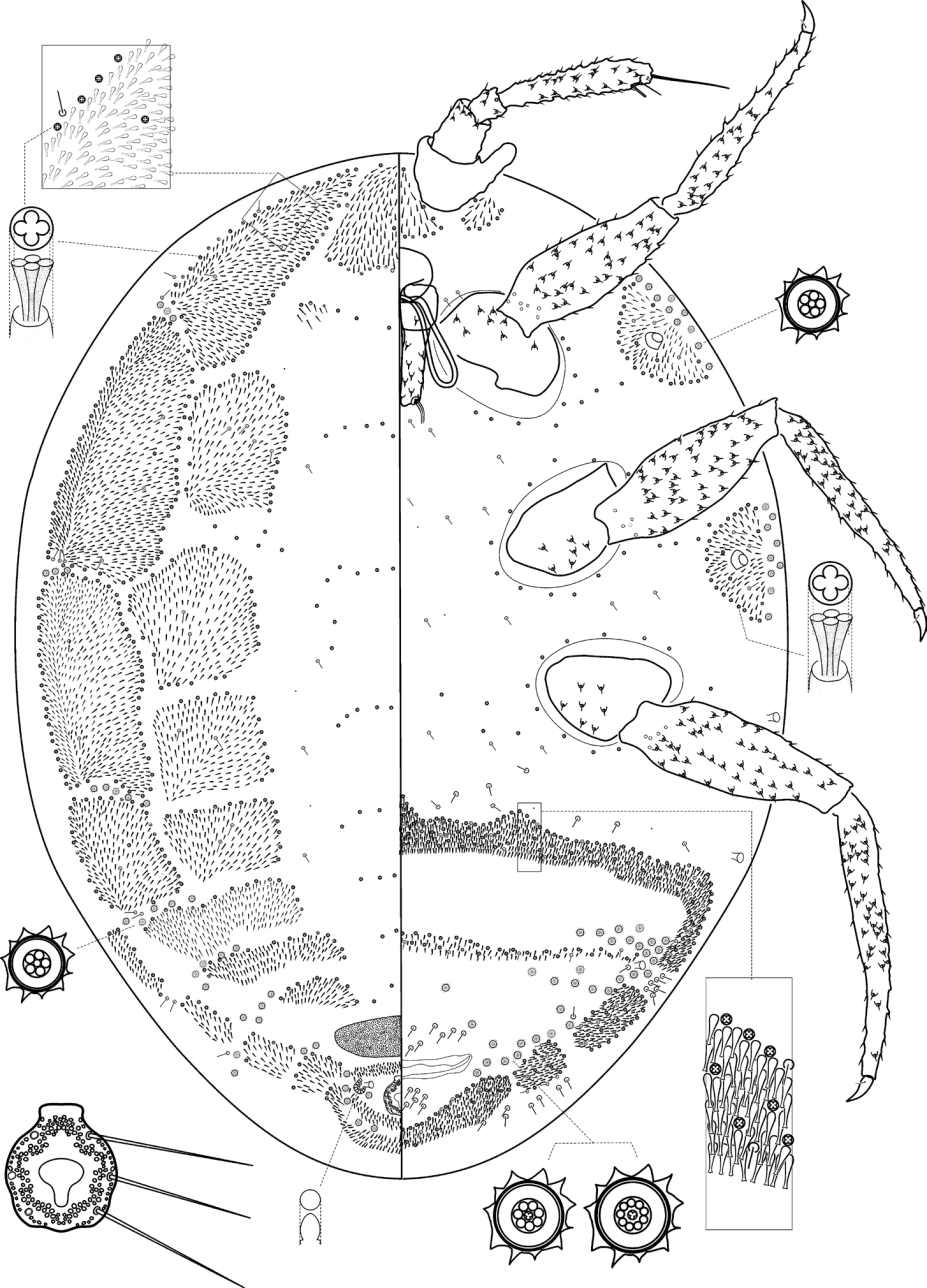
*Ortheziola editae* Szita & Konczné Benedicty, sp. n., adult female, holotype.

##### Host plant.

Unknown.

##### Distribution.

Bulgaria, Croatia.

##### Etymology.

The new species is dedicated to Edit Horváth, who has worked as an assistant in the Acarology Collection of the Hungarian Natural History Museum, Budapest for many years and helped our work in extracting the specimens and finding locality data.

##### Comments.

*Ortheziola editae* is characterized by the presence of (i) dorsal wax plate 3 being only slightly developed, (ii) ventral plates 11 and 12 present at the base of antennae, and (iii) plate 19 absent from near the body margin. This species is very close to *Ortheziola marottai* but differs in having (*Ortheziola marottai* values in brackets): (i) only one or two multilocular pores anterior to vulva (plenty of multilocular pores); (ii) multilocular pores near each thoracic spiracle, each pore with five loculi (four loculi) and (iii) anterior margin of ovisac band with six waves (at least eight waves).

#### Ortheziola marottai

Taxon classificationAnimaliaHemipteraOrtheziidae

Kaydan & Szita

##### Material examined.

Greece: 1 ♀ – Ioannina regional unit, Kalpaki, Vellas Monasteri. Macedonia: 2 ♀♀ – Prilep Municipality, Raec canyon. Romania: 3 ♀♀ – Alba County, Munții Apuseni Mts., Cheile Albioarei, Tarina village; 1 ♀ – Hunedoara County, Retyezát Mts., Campu lui Neag village.

##### Distribution.

Croatia (former Yugoslavia), Cyprus, Greece, Iran, Turkey (Kaydan et al. 2014); **Macedonia, Romania**.

#### Ortheziola vejdovskyi

Taxon classificationAnimaliaHemipteraOrtheziidae

Šulc

##### Material examined.

Bosnia-Herzegovina: 1 ♀ – Prenj Mts., Borci. Croatia: 1 ♀ – Krapina Zagorje County, Ivansaica Mts., Stari Golubovec; 4 ♀♀ – Mala-kapela, Plitvice Lakes; 2 ♀ – Papuk, Štrmac. France: 1 ♀ – Midi Pyrenees, Arreau. Italy: 1 ♀ – Abruzzi, Mts. Maiella, Sulmona. Romania: 1 ♀ – Alba County, Runc (Aranyosronk), Runki-szoros; 6 ♀ – Alba County, Rimetea (Torockó);1 ♀ – Bihor County, Bihor Mts., Vislo village; 1 ♀ – Bukovina County, Voievodeasa; 1 ♀ – Caraş-Severin County, Ţarcu Mts., Poiana Mărului; 1 ♀ – Cluj County, Sinfalva, Aranyos valley; 1 ♀ – Cluj County, Turda (Torda), Cheile Turzii (Tordai hasadék); 2 ♀ – Harghita County, Băile Homorod (Homoródfürdő); 1 ♀ – Maramureş County, Maramureş Mts., Petrova, Frumuena; 1 ♀ – Maramureş County, Baia Mare (Nagybánya), Valhani plateau, Rozsály Mt.; 1 ♀ – Maramureş County, Rodna Mts., Săcel (Izaszacsal); 1 ♀ – Maramureş County, Săpânţa (Szaplonca), Kőhát; 1 ♀ – Maramureş County, Sighetu Marmatiei; 1 ♀ – Satu Mare County, Negrești-Oaș. Russia: 1 ♀ – Chechnya, Dzheirakhs District, Olgeti village. Serbia-Montenegro: 1 ♀ – Savino Polje; 1 ♀ – Zlatibor Mts., Vodice. Slovakia: 2 ♀ – Červený Kláštor, Pieniny National Park; 1 ♀ – Košice (Kassa); 4 ♀ – Slovakian Raj NP, Cingov; 1 ♀ – Staré Hory (Óhegy). Slovenia: 1 ♀ – Bohinjska Bela; 1 ♀ – Predjama; 1 ♀ – Ribcev Laz Lake Bohijsko Jezero; 1 ♀ – Triglav NP., Koča pri Peričniku. Ukraine: 1 ♀ – Kiev.

##### Distribution.

Armenia, Austria, Azores, Belgium, China (Beijing (=Peking)), Corsica, former Czechoslovakia, France, Germany, Hungary, Italy, Luxembourg, Madeira Islands, Netherlands, Poland, Romania, Sweden, Switzerland, USSR, Ukraine, United Kingdom (England, Scotland, Wales), former Yugoslavia (García et al. 2015); Bosnia-Herzegovina, Croatia, Montenegro, Serbia, **Slovakia**, Slovenia.

##### Comments.

The type locality of *Ortheziola vejdovskyi* is in Czech Republic, originally: Bohemia, Bechlin; Králové Dvur n. L. east Bohemi (Šulc 1895), and this is the only report from the area of former Czechoslovakia (García et al. 2015), thus the current data from Slovakia can be considered as a new country record. *Ortheziola vejdovskyi* was reported from Yugoslavia by Kosztarab and Kozár (1988) (García et al. 2015), without detailed locality data, therefore we have no exact information which current successor state(s) could have been the actual locality(ies) in that report. Accordingly we list the current localities by states, without considering these as new country records, thus it was not unequivocally proven.

### Pseudococcidae

#### Atrococcus parvulus

Taxon classificationAnimaliaHemipteraPseudococcidae

(Borchsenius)

##### Material examined.

Slovakia: 1 ♀ – Pieniny National Park, Červený Kláštor.

##### Distribution.

China, Kazakhstan, Kyrgyzstan, Tajikistan, Turkey, Uzbekistan (García et al. 2015); **Slovakia**.

#### Ferrisia malvastra

Taxon classificationAnimaliaHemipteraPseudococcidae

(McDaniel)

##### Material examined.

Spain: 1 ♀ – Canary Island, Tenerife, Masca.

##### Distribution.

Argentina, Ascension Island, Australia (Queensland), Bahamas, Bermuda, Brazil, Canary Islands, Cook Islands, Cuba, Hawaiian Islands (Hawaii), India, Israel, Jamaica, Kiribati, Mexico, New Caledonia, Papua New Guinea, Peru, South Africa, Spain, Sri Lanka, Swaziland, Tobago, Tonga, Trinidad, Tuvalu, United States of America, Vanuatu, Venezuela (García et al. 2015).

#### Balanococcus boratynskii

Taxon classificationAnimaliaHemipteraPseudococcidae

Williams

##### Material examined.

Romania: 1 ♀ – Maramureş County, Maramureş Mts., Borşa-Băile Borşa.

##### Distribution.

Bulgaria, Hungary, Italy, Poland, Russia, Sweden, Switzerland, United Kingdom (England) (García et al. 2015); **Romania**.

#### Balanococcus orientalis

Taxon classificationAnimaliaHemipteraPseudococcidae

Danzig & Ivanova

##### Material examined.

Albania: 1 ♀ – Shkodër Municipality, Shkodër, Castle of Rozafat. Romania: 1 ♀ – Maramureş County, Maramureş Mts., Borşa-Băile Borşa.

##### Distribution.

Italy, North Korea, Russia, Sardinia (García et al. 2015); **Albania, Romania**.

#### Genus Brevennia

Taxon classificationAnimaliaHemipteraPseudococcidae

Goux

Ripersia Goux, 1940: 58. Type species: *Ripersia tetrapora* Goux by original designation. Accepted valid name.
Asphodelococcus Morrison, 1945: 41. Type species: *Ripersia asphodeli* Bodenheimer by monotypy and original designation. Junior synonym.
Brevennia Borchsenius, 1948: 953. Change of status.
Asphodeloripersia Bodenheimer, 1953: 164. Misspelling of genus name.
Pseudorhodania Borchsenius, 1962: 242. Type species: *Pseudorhodania marginata* Borchsenius, by original designation. Synonymy by Danzig and Gavrilov-Zimin 2012a: 786.

##### Type species.

Ripersia (Brevennia) tetrapora Goux, 1940: 58.

##### Diagnosis.

*Living female*. Female covered with white wax powder.

*Adult female*. Labium three-segmented, longer than wide. Posterior pair of spiracles always larger than anterior spiracles. Circulus present or absent. Legs well developed, claw with or without denticle; tarsal digitules hair-like, not capitate; claw digitules knobbed, claw digitules broader than tarsal digitules. Only posterior ostioles developed; anterior ostioles absent. Anal lobes poorly developed. Anal ring oval, with one inner row of pores and one or two outer rows of pores plus with six setae. Minute discodial pores present of various sizes, scattered throughout.

*Dorsum*. Antennae 6-8 segmented. Eyes oval, each on a small basal cone. Cerarii present numbering 1-4, only on posterior abdominal segments. Dorsal body setae spinelike. Multilocular disc pores present or absent. Quinquelocular pores present, scattered all surface. Oral collar tubular present in transverse rows on body segments. Trilocular pores absent. Minute discodial pores present, from a few to scattered on the surface, variable in sizes.

*Venter*. Most ventral setae slender and hair-like, of various sizes. Oral collar tubular ducts of one or two sizes, each varying in length and width. Multilocular disc pores present on posterior abdominal segments, especially around vulva or absent. Quinquelocular pores present, scattered throughout. Trilocular pores, each 2.5–5.0 μm in diameter, only around atrium of both pairs of spiracles. Minute discodial pores present, of variable sizes, scattered through.

*Comments*. In this study, the concept of Kaydan (2011) and Foldi and Cox (1989) are accepted and *Brevennia* Goux *sensu stricto* is regarded as a valid genus and is considered to include: *Brevennia cicatricosa* (Danzig), *Brevennia dasiphorae* (Danzig), *Brevennia filicta* (De Lotto), *Brevennia oryzae* (Tang), *Brevennia pulveraria* (Newstead) and *Brevennia rehi* (Borchsenius). These species are characterized by: (i) lack of anterior ostioles; (ii) trilocular pores restricted to around each spiracular atrium on the venter and to the cerarii on the dorsum. For further discussion see Danzig and Gavrilov (2012; 2013), Kaydan (2011) and Foldi and Cox (1989).

##### Key to adult female *Brevennia*

(adapted from Danzig and Gavrilov 2012)

**Table:** 

1	Multilocular pores present either on venter or dorsum	**2**
–	Multilocular pores absent from both venter and dorsum	***Brevennia larvalis* sp. n.**
2	Multilocular pores absent on dorsum	**3**
–	Multilocular pores present on dorsum	**4**
3	Trilocular pores situated in cerarii and near spiracles; one circulus present	***Brevennia cicatricosa* (Danzig)**
–	Trilocular pores situated only in cerarii; circuli absent	***Brevennia dasiphorae* (Danzig)**
4	Cerarii with quinquelocular pores only	**5**
–	Cerarii with both quinquelocular pores and trilocular pores	**6**
5	Multiocular disc pores on dorsum present on margin of head, thorax and abdominal segments	***Brevennia rehii* (Maxwell-Lefroy)**
–	Multiocular disc pores on dorsum present only on margin of abdominal segments	***Brevennia oryzae* (Tang)**
6	Multilocular disc pores on dorsum wide band on body margin and present on mid-abdominal area of posterior abdominal segments	***Brevennia filicta* (De Lotto)**
–	Multilocular disc pores on dorsum few on body margin and absent on mid-abdominal area of posterior abdominal segments	***Brevennia pulveraria* (Newstead)**

#### Brevennia larvalis

Taxon classificationAnimaliaHemipteraPseudococcidae

Kaydan sp. n.

http://zoobank.org/A63FA89F-F938-4E9E-ACEB-01F4307AFE91

Figs 3 , 4

##### Material examined.

*Holotype*. Adult female. Albania: Qafa e Pejës, 1700 m a.s.l., 17.vii.1996, leg. Horváth E. (PPI: 12211, HNHM: E-1451). *Other material examined*. 5 nymphs – same data as holotype.

##### Description.

**Figure 3.**
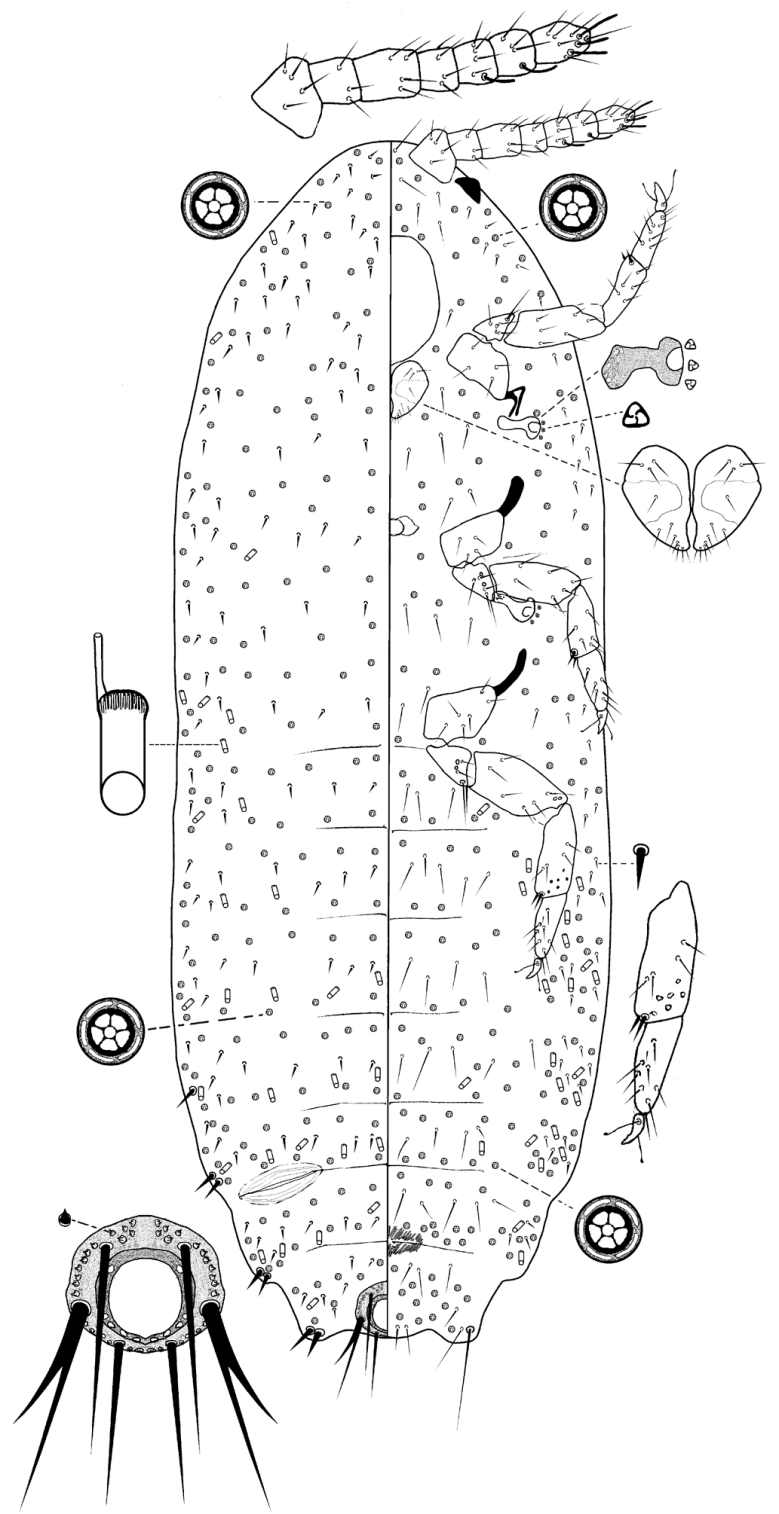
*Brevennia larvalis* Kaydan, sp. n., adult female, holotype.

*Adult female* (Fig. 3). Body elongate oval, 1.24 mm long, 0.48 mm wide. Eye marginal, 35–40 µm wide. Antenna seven or eight segmented, 1.90 µm long; apical segment 32.5–35 µm long, 25–30 µm wide, with apical setae 22.5–27.5 µm long plus three fleshy setae, each 20–35 µm long. Tentorium 135 µm long, 120 µm wide. Labium 65 µm long, 90 µm wide. Anterior spiracles 37.5–42.5 µm long, 17.5–20.0 µm wide across atrium; posterior spiracles 45 µm long, 22.5–25.0 µm wide across atrium; each spiracle associated with 2 or 3 trilocular pores. Legs well developed; data for posterior legs: coxa 70 µm, trochanter + femur 125 µm, tibia + tarsus 135 µm, claw 17.5 µm. Ratio of lengths of tibia + tarsus to trochanter + femur 1.02–1.70:1; ratio of lengths of tibia to tarsus 1.23–1.70:1; ratio of length of hind trochanter + femur to greatest width of femur 3.45–3.80:1. Tarsal digitules each 25 µm long, hair-like. Claw digitules knobbed each 17.5 µm long. Hind tibia with 4–9 translucent pores. Anterior ostioles absent; posterior ostioles present, without pores or setae. Anal ring 60 µm wide, with six setae, each seta 55–90 µm long. Cerarii three pairs only, each slightly sclerotized; anal lobe cerarii each with two enlarged setae, 15 µm long, plus one quiquelocular pore; cerarii on abdominal segments VII and VI both with two slender enlarged setae and two or three quinquelocular pores.

*Dorsum*. Body setae spine-like in various sizes, each 5.0–12.5 µm long. Quinquelocular pores in rows on abdominal segments as follows: I-III 84, IV 32, V 32, VI 39, VII 34, VIII + IX 11; each pore 5–6 µm in diameter; pores scattered on head and thorax. Oral collar tubular ducts, each 7.5–10 µm long, 4–5 µm wide, in single rows across all abdominal segments: I-III 14 ducts, IV 8, V 8, VI 10, VII 9, VIII + IX 3, and also submarginal area of head and thorax, each pore 5–6 µm in diameter. Minute discoidal pores scattered throughout, each 2 µm in diameter.

*Venter*. Setae slender, hair-like, each 10–35 µm long, longest setae medially on head. Apical setae of anal lobe each 110–120 µm long. Multilocular disc pores absent. Quinquelocular pores each 5–6 µm in diameter; in rows on abdominal segments as follows: II-III 74, IV 36, V 45, VI 39, VII 50, VIII + IX 34; and scattered on head and thorax. Minute discoidal pores few, each 2 µm in diameter, scattered throughout. Oral collar tubular ducts concentrated on body margin of abdominal segments, of one size, each 4–5 µm long, 7.5–10 µm wide, and on margin of head, thorax and abdominal segments, as follows: II-III 30 ducts, IV 12, V 10, VI 9, VII 4, VIII + IX.

##### Comments.

*Brevennia larvalis* sp. n. Kaydan can be readily distinguished by: (i) absence of multilocular pores; (ii) absence of pores and setae on the lips of ostioles; and (iii) in having three pairs of cerarii. There is no other species in the genus without multilocular pores.

**Figure 4.**
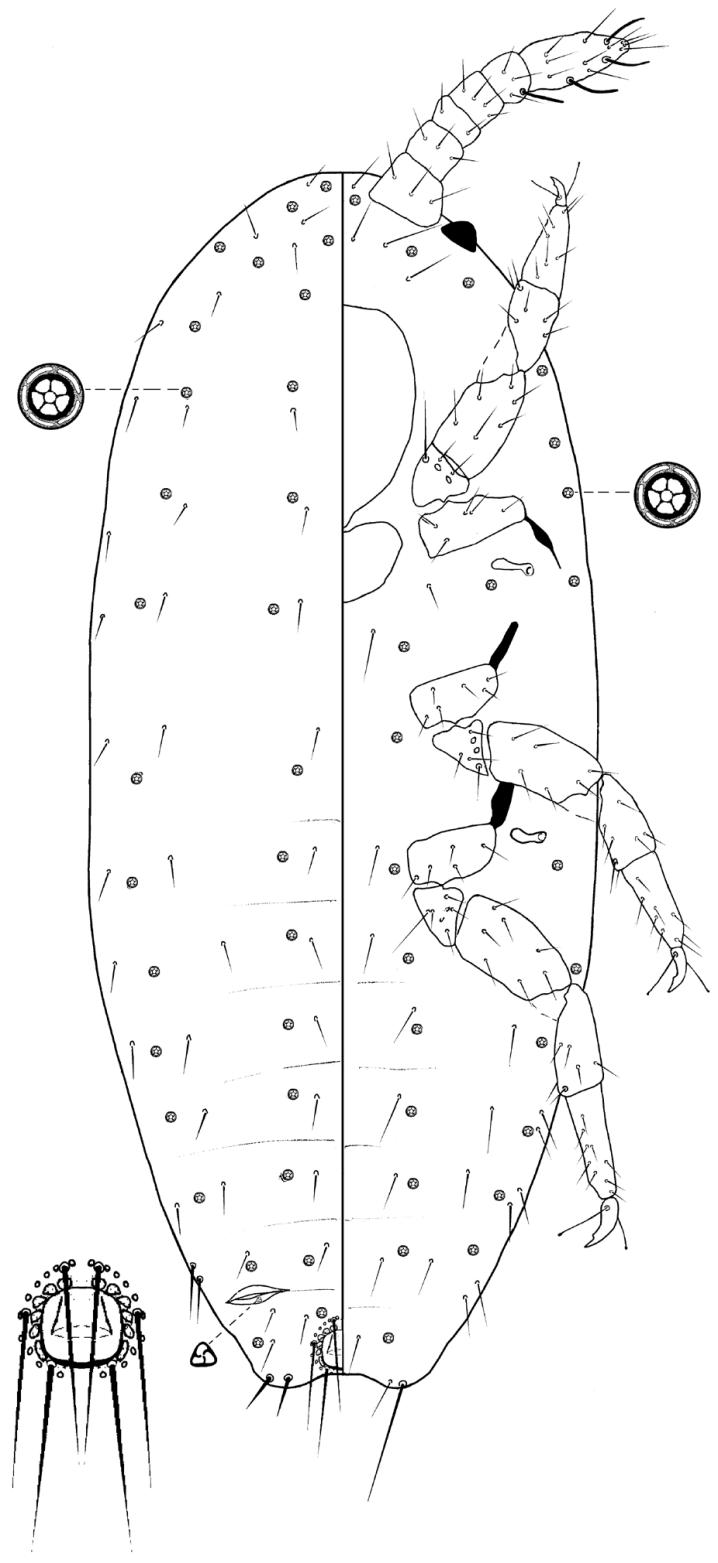
*Brevennia larvalis* Kaydan, sp. n., first instar nymph.

*First-instar nymph* (Fig. 4). Body elongate oval, 0.51–0.56 mm long, 0.20–0.22 mm wide. Eye marginal, 35–40 µm wide. Antenna six-segmented, 1.30–1.90 µm long; apical segment 45–52.5 µm long, 22.5–27.5 µm wide, with apical setae 22.5–27.5 µm long plus three fleshy setae, each 15–17.5 µm long. Tentorium 80 µm long, 75 µm wide. Labium 40-45 µm long, 52.5 µm wide. Anterior spiracles 22.5–25 µm long, 7.5 µm wide across atrium; posterior spiracles 22.5–25 µm long, 7.5 µm wide across atrium. Legs well developed; data for posterior legs: coxa 37.5–42.5 µm, trochanter + femur 75.0–82.5 µm, tibia + tarsus 92.5–95 µm, claw 12.5–15.0 µm. Ratio of lengths of tibia + tarsus to trochanter + femur 1.02–1.70:1; ratio of lengths of tibia to tarsus 1.23–1.70:1; ratio of length of hind trochanter + femur to greatest width of femur 3.45–3.80:1. Tarsal digitules each 15–20 µm long, hair-like. Claw digitules knobbed each 12.5–15 µm long. Anterior ostioles absent; posterior ostioles present with only one trilocular pore 2.5–3.0 µm in diameter. Anal ring 42.5 µm wide, with six setae, each seta 40 µm long. Cerarii two pairs only; anal lobe cerarii each with two enlarged setae, 15–25 µm long, cerarius on abdominal segment VII with two slender enlarged setae.

*Dorsum*. Body setae spinelike of various sizes, each 5.0–12.5 µm long. Quinquelocular pores in four longitudinal rows, each pore 5–6 µm in diameter.

*Venter*. Setae slender and hair-like, each 15–25 µm long, longest setae medially on head. Apical setae of anal lobe each 42.5–85.0 µm long. Quinquelocular pores in four longitudinal rows, each pore 5–6 µm in diameter.

##### Etymology.

This species is named because of absence of multilocular pores on venter and dorsum, which is a character of larval (nymphal) stages.

##### Host plants.

Unknown.

##### Distribution.

Albania.

#### Fonscolombia europaea

Taxon classificationAnimaliaHemipteraPseudococcidae

(Newstead)

##### Material examined.

Greece: 3 ♀♀ – Epirus, Ioannina regional unit, Lakmos Mts. Romania: 6 ♀♀ – Cluj County, Cheile Turzii (Tordai hasadék).

##### Distribution.

Armenia, Austria, France, Germany, Hungary, Italy, Luxembourg, Mongolia, Netherlands, Poland, Russia, Sweden, Turkey, Ukraine, United Kingdom (Channel Islands, England) (García et al. 2015); **Greece, Romania**.

#### Fonscolombia graminis

Taxon classificationAnimaliaHemipteraPseudococcidae

Lichtenstein

##### Material examined.

Croatia: 1♀ – Njivice.

##### Distribution.

Corsica; France (García et al. 2015); **Croatia**.

#### Metadenopus festucae

Taxon classificationAnimaliaHemipteraPseudococcidae

Šulc

##### Material examined.

Greece: 1♀, 1 nymph – West Greece, Aetolia-Acarnania regional unit, Panetoliko Mts., Agios Vlasios.

##### Distribution.

China, Czech Republic, France, Hungary, Italy, Moldova, Mongolia, Poland, Russia, Turkey, Ukraine (García et al. 2015); **Greece**.

#### Mirococcopsis subterranea

Taxon classificationAnimaliaHemipteraPseudococcidae

(Newstead)

##### Material examined.

Romania: 4 ♀♀ – Cluj County, Cheile Turzii (Tordai hasadék).

##### Distribution.

Armenia, Czech Republic, Denmark, France, Georgia (Georgia) , Hungary, Italy, Kazakhstan, Lithuania, Netherlands, Poland, Russia, Spain, Sweden, Ukraine, United Kingdom (Channel Islands, England, Scotland) (García et al. 2015); **Romania**.

#### Peliococcus chersonensis

Taxon classificationAnimaliaHemipteraPseudococcidae

(Kiritshenko)

##### Material examined.

Bulgaria: 1♀ – Plovdiv Province, Asenovgrad.

##### Distribution.

Armenia, China; Italy, Kazakhstan, Lithuania, Mongolia, Russia, South Korea, Turkey, Ukraine (García et al. 2015); **Bulgaria**.

#### Peliococcus loculatus

Taxon classificationAnimaliaHemipteraPseudococcidae

Danzig

##### Material examined.

Romania: 1 ♀ – Maramureş County, Rodna Mts., Borşa-Staţiunea Borşa.

##### Distribution.

Russia (García et al. 2015); **Romania**.

#### Pelionella manifecta

Taxon classificationAnimaliaHemipteraPseudococcidae

(Borchsenius)

##### Material examined.

Greece: 1 ♀ – Kos.

##### Distribution.

Armenia, Azerbaijan, Italy, Kazakhstan, Sardinia, Sweden, Turkey (García et al. 2015); **Greece**.

#### Phenacoccus abditus

Taxon classificationAnimaliaHemipteraPseudococcidae

Borchsenius

##### Material examined.

Croatia: 1 ♀ – Njivice.

##### Distribution.

Armenia, Crete, Georgia, Hungary, Kazakhstan, Poland, Russia, Tajikistan, Turkey, Turkmenistan (García et al. 2015); **Croatia**.

#### Phenacoccus hordei

Taxon classificationAnimaliaHemipteraPseudococcidae

(Lindeman)

##### Material examined.

Albania: 2 ♀♀ –Mat District, Qafa e Shtamës.

##### Distribution.

Armenia, Finland, France, Germany, Greece, Hungary, Iran, Italy, Kazakhstan, Moldova, Netherlands, Poland, Russia, Sweden, Turkey, Ukraine, United Kingdom (England) (García et al. 2015); **Albania**.

#### Phenacoccus karaberdi

Taxon classificationAnimaliaHemipteraPseudococcidae

Borchsenius & Ter-Grigorian

##### Material examined.

Greece: 3 ♀♀ – Epirus, Ioannina regional unit, Lakmos Mts.

##### Distribution.

Armenia, Austria, Kazakhstan, Russia, Tajikistan, Turkey (García et al. 2015); **Greece**.

#### Phenacoccus poriferus

Taxon classificationAnimaliaHemipteraPseudococcidae

Borchsenius

##### Material examined.

Serbia: 1 ♀ – Niš.

##### Distribution.

China, Mongolia, North Korea, Russia, Tajikistan (García et al. 2015), **Serbia**.

#### Phenacoccus specificus

Taxon classificationAnimaliaHemipteraPseudococcidae

Matesova

##### Material examined.

Greece: 1 ♀, 1 nymph – Pieria regional unit, Olympos, Litochoro.

##### Distribution.

Kazakhstan (García et al. 2015); **Greece**.

#### Phenacoccus tergrigorianae

Taxon classificationAnimaliaHemipteraPseudococcidae

Borchsenius

##### Material examined.

Greece: 1 ♀ – West Greece, Aetolia-Acarnania regional unit, Panetoliko Mts., Agios Vlasios village.

##### Distribution.

Armenia, Turkey (García et al. 2015), **Greece**.

#### Rhodania porifera

Taxon classificationAnimaliaHemipteraPseudococcidae

Goux

##### Material examined.

Bulgaria: 2 ♀♀ – Belogradchik.

##### Distribution.

Armenia, France, Georgia, Germany, Hungary, Italy, Kazakhstan, Mongolia, Poland, Russia, Turkey, Ukraine (García et al. 2015); **Bulgaria**.

#### Trionymus newsteadi

Taxon classificationAnimaliaHemipteraPseudococcidae

(Green)

##### Material examined.

Slovakia: 1 ♀ – Štos-Kupele.

##### Distribution.

Armenia, Czech Republic, Germany, Hungary, Italy, Netherlands, Poland, Russia, Ukraine, United Kingdom (England), former Yugoslavia (García et al. 2015); **Slovakia**.

#### Volvicoccus volvifer

Taxon classificationAnimaliaHemipteraPseudococcidae

(Goux)

##### Material examined.

Romania: 1♀ – Cluj County, Cheile Turzii (Tordai hasadék).

##### Distribution.

Armenia, Bulgaria, France, Hungary, Italy, Poland, Turkey, Ukraine (García et al. 2015); **Romania**.

### Putoidae

#### Puto antennatus

Taxon classificationAnimaliaHemipteraPutoidae

(Signoret)

##### Material examined.

Bulgaria: 1 ♀ –Pirin, Demianitsa; 1 ♀ – Pirin; Vihren; 1 ♀ – Vitosha. Serbia: 1 ♀ – Savino Polje, Đalovica klisura.

##### Distribution.

Austria, Bulgaria, Czech Republic, France, Germany, Italy, Serbia, Switzerland (García et al. 2015).

##### Comments.

Occurring on needles and in bark crevices of conifers. Biology in Italy studied by Sampo and Olmi (1979). Life history discussed by Kosztarab and Kozár (1988).

### Rhizoecidae

#### Rhizoecus albidus

Taxon classificationAnimaliaHemipteraRhizoecidae

Goux

##### Material examined.

Romania: 1 ♀ – Prahova County, Cheia Cul. Mea, Gropsoarale, Zagram.

##### Distribution.

Armenia, Crete, France, Germany, Hungary, Iran, Italy, Kazakhstan, Romania, Russia, Sweden, Ukraine, United Kingdom (England) (García et al. 2015).

#### Rhizoecus kazachstanus

Taxon classificationAnimaliaHemipteraRhizoecidae

Matesova

##### Material examined.

Albania: 1 second instar nymph – Skrapar District, Tomor Mts., Skrapar.

##### Distribution.

Albania, Hungary, Kazakhstan (García et al. 2015).

#### Rhizoecus pseudocacticans

Taxon classificationAnimaliaHemipteraRhizoecidae

Hambleton

Fig. 5

##### Material examined.

Spain: 1 ♀ – Canary Islands, Tenerife, Masca, 450 m a.s.l., 20.x.2008, leg. Jely Z., soil (PPI: 11938, HNHM: E-2531).

##### Host plants.

*Crassula* sp., *Kalanchoe tomentosa*, *Sedum* sp. (Crassulaceae), *Aloe* sp. (Liliaceae) (García et al. 2015).

##### Distribution.

United States of America (García et al. 2015), **Spain**.

##### Comment.

This species is characterized by the lack of multilocular pores on both the dorsum and venter and in having very few oral collar tubular ducts on the dorsum. This species is similar to *Rhizoecus cacticans* and *Rhizoecus leucosomus*, but differs from both in having more anal ring pores. In addition, this species is also similar to *Rhizoecus nakaharai* but differs in having a longer labium. However, these are poor characteristics upon which to base species differences and so a drawing of this species is presented here. Detailed descriptions of the above mentioned species are available in Kozár and Konczné Benedicty (2007).

**Figure 5.**
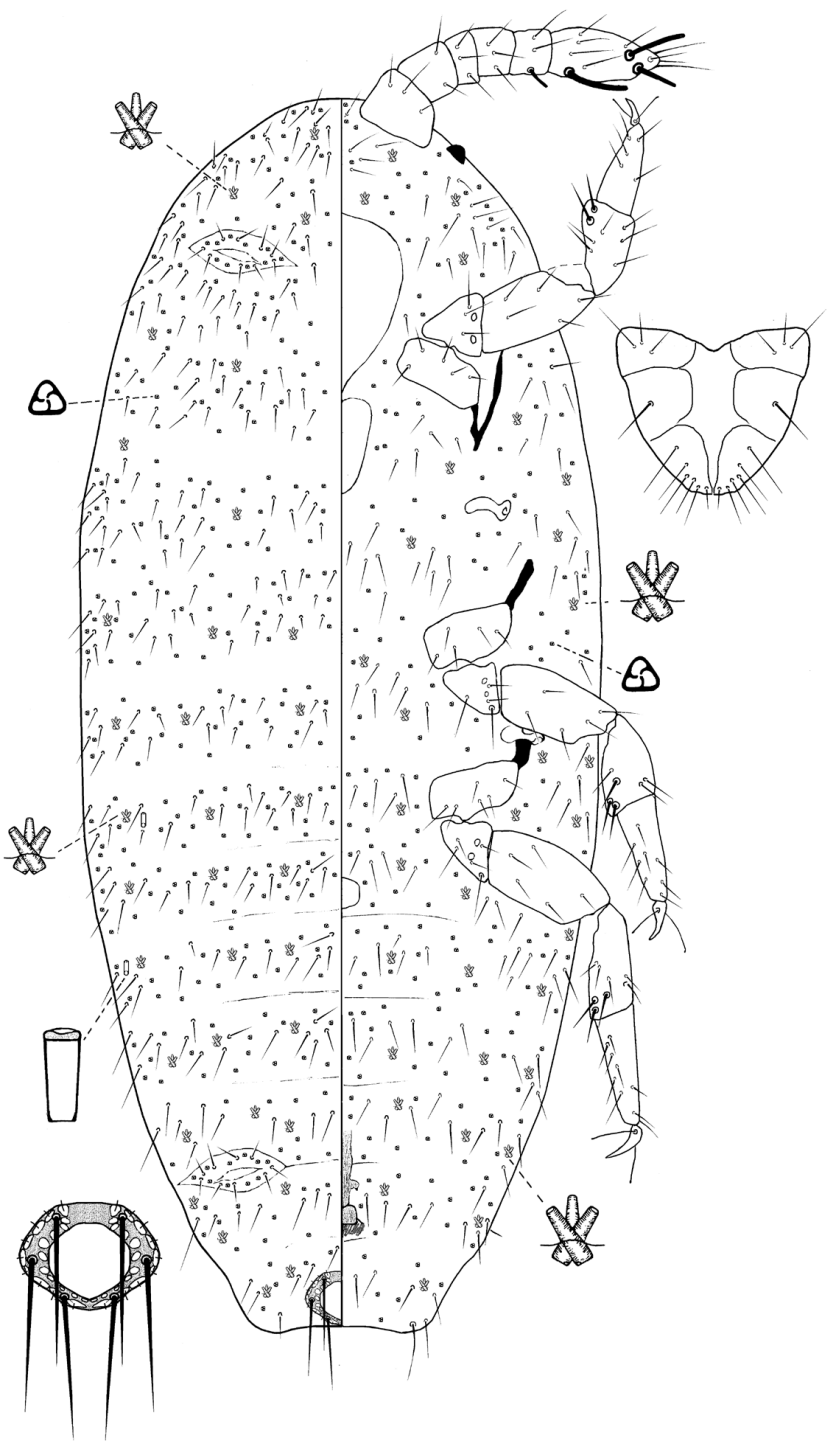
*Rhizoecus pseudocacticans*, Hambleton, original.

#### Ripersiella caesii

Taxon classificationAnimaliaHemipteraRhizoecidae

(Schmutterer)

##### Material exmined.

Serbia: 2 ♀♀ – Niš; 5 ♀♀ – Špiljani.

##### Distribution.

Germany (García et al. 2015); **Serbia**.

#### Ripersiella halophila

Taxon classificationAnimaliaHemipteraRhizoecidae

(Hardy)

##### Material exmined.

Albania: 2 ♀♀ – Kukës District, Topojan.

##### Distribution.

Bulgaria, Czech Republic, France, Germany, Hungary, Ireland, Poland, Russia, Ukraine, United Kingdom (England, Scotland, Wales) (García et al. 2015); **Albania**.

#### Ripersiella parva

Taxon classificationAnimaliaHemipteraRhizoecidae

(Danzig)

##### Material examined.

Albania: 6 nymphs – Librazhd District, Gizavësh, Librazhd; 2 nymphs – Mirditë District, Ndërshenë; 1 nymph – Mat District, Dejë Mts., Macukull; 25 nymphs – Pogradec District, Lin.

##### Distribution.

Albania, Russia, Turkey (García et al. 2015).

#### Ripersiella periolana

Taxon classificationAnimaliaHemipteraRhizoecidae

Goux

##### Material examined.

Greece: 1 ♀, 1 nymph – Pieria regional unit, Olympos Mts., Litochoro.

##### Distribution.

Greece, Hungary, Italy, Turkey (García et al. 2015).

## Discussion

In this study, 43 scale insect species were found in 16 different European countries. Despite scale insects being found in only 10% of the 2970 samples collected, the Berlese funnel collection method has revealed new species and widened distribution records for known species. It is believed that the use of diverse collecting methods can provide researchers with additional sources of information about species distribution and diversity.

## Supplementary Material

XML Treatment for Anophococcus insignis

XML Treatment for Kaweckia glyceriae

XML Treatment for Pseudochermes fraxini

XML Treatment for Rhizococcus reynei

XML Treatment for Lecanopsis turcica

XML Treatment for Luzulaspis dactylis

XML Treatment for Micrococcus confusus

XML Treatment for Arctorthezia cataphracta

XML Treatment for Arctorthezia helvetica

XML Treatment for Newsteadia floccosa

XML Treatment for Newsteadia susannae

XML Treatment for Ortheziola

XML Treatment for Ortheziola editae

XML Treatment for Ortheziola marottai

XML Treatment for Ortheziola vejdovskyi

XML Treatment for Atrococcus parvulus

XML Treatment for Ferrisia malvastra

XML Treatment for Balanococcus boratynskii

XML Treatment for Balanococcus orientalis

XML Treatment for Brevennia

XML Treatment for Brevennia larvalis

XML Treatment for Fonscolombia europaea

XML Treatment for Fonscolombia graminis

XML Treatment for Metadenopus festucae

XML Treatment for Mirococcopsis subterranea

XML Treatment for Peliococcus chersonensis

XML Treatment for Peliococcus loculatus

XML Treatment for Pelionella manifecta

XML Treatment for Phenacoccus abditus

XML Treatment for Phenacoccus hordei

XML Treatment for Phenacoccus karaberdi

XML Treatment for Phenacoccus poriferus

XML Treatment for Phenacoccus specificus

XML Treatment for Phenacoccus tergrigorianae

XML Treatment for Rhodania porifera

XML Treatment for Trionymus newsteadi

XML Treatment for Volvicoccus volvifer

XML Treatment for Puto antennatus

XML Treatment for Rhizoecus albidus

XML Treatment for Rhizoecus kazachstanus

XML Treatment for Rhizoecus pseudocacticans

XML Treatment for Ripersiella caesii

XML Treatment for Ripersiella halophila

XML Treatment for Ripersiella parva

XML Treatment for Ripersiella periolana
